# Strategies to Improve γ-Aminobutyric Acid Biosynthesis in Rice via Optimal Conditions

**DOI:** 10.3390/plants14091290

**Published:** 2025-04-24

**Authors:** Lixing Shen, Yulu Yang, Xiong Liu, Huibo Zhao, Yanfang Zhang, Lan Shen, Li Zhu, Jiang Hu, Deyong Ren, Qiang Zhang, Zhenyu Gao, Guojun Dong, Qing Li, Qian Qian, Dali Zeng, Guangheng Zhang

**Affiliations:** 1 College of Advanced Agricultural Sciences, Zhejiang A&F University, Hangzhou 311300, China; 17857367023@163.com; 2 State Key Laboratory of Rice Biology and Breeding, China National Rice Research Institute, Hangzhou 310006, China; yulu97_y@163.com (Y.Y.); liu18370815743@163.com (X.L.); zhaohuibo000@163.com (H.Z.); 15009657195@163.com (Y.Z.); baishushenlan@126.com (L.S.); zhuli05@caas.cn (L.Z.); hujiang588@163.com (J.H.); rendeyong616@163.com (D.R.); zhangqiang9024@126.com (Q.Z.); gaozhenyu@caas.cn (Z.G.); dongguojun@caas.cn (G.D.); liqing1986102@163.com (Q.L.); qianqian@hotmail.com (Q.Q.); 3 National Nanfan Research Institute (Sanya), Chinese Academy of Agricultural Sciences, Sanya 572024, China

**Keywords:** rice (*Oryza sativa* L.), embryo, γ-aminobutyric acid (GABA), germinated, enrichment conditions, amino acid content

## Abstract

The γ-aminobutyric acid (GABA), a ubiquitous non-protein amino acid in plants and animals, exhibits diverse biological activities and holds promise in human disease prevention and treatment. Prior studies have shown that germination could substantially elevate GABA levels in rice, but these investigations typically focused on limited germplasms, hindering the generalization of their findings. This study aims to identify optimal conditions for enriching GABA in a diverse set of 225 rice germplasms by examining the effects of various germination times, temperatures, and soaking solution pH levels, while elucidating the key factors influencing GABA enrichment in germinated brown rice. The optimal GABA enrichment in germinated brown rice was achieved under the following conditions: a germination temperature of 37 °C, a germination duration of 48 h, and a soaking solution pH of 5.5. Under these conditions, we found significant differences in GABA content among different germplasms. Subsequent correlation analyses demonstrated that GABA content showed significant positive correlations with embryo weight in brown rice, relative embryo weight, relative embryo weight in germinated brown rice, as well as glutamate (Glu) and proline (Pro) concentrations. Therefore, larger brown rice embryos, higher Glu and Pro content in germinated brown rice, and external Glu application contribute to increased GABA content. Our findings provide essential materials and theoretical insights for screening and developing GABA-rich functional rice germplasms, facilitating variety selection and breeding programs.

## 1. Introduction

γ-aminobutyric acid (GABA) is a non-protein amino acid synthesized through two pathways: the GABA biosynthesis pathway and polyamine degradation pathway. It is ubiquitously found in microorganisms, plants, and animals. GABA exhibits a variety of biological activities, playing a crucial role in protecting the nervous system and preventing related diseases, such as hypertension, diabetes, oxidation, and promoting sleep [1,2]. Consequently, the development of GABA-rich foods or health products has become a focal point. Cereal grains, particularly rice, wheat, and maize, have been essential components of the human diet for millennia, providing vital energy and nutrients like proteins, vitamins, and minerals [3]. These cereals are significant sources of GABA [4]. Rice, in particular, serves as the primary food source for over half of the global population. The use of brown rice in food production has garnered increasing attention due to its nutritional benefits [5]. This has led to the development of a wide range of commercialized, germinated brown rice products, including rice noodles and yogurt, which have experienced rapid growth in the global market. Ingesting GABA from natural foods, such as rice, is a safer approach. Therefore, enriching GABA in foods using various techniques is more acceptable than adding GABA directly as a food additive [6]. Studies have shown that germinated brown rice contains significantly higher levels of amino acids compared to ungerminated brown rice, with up to three times the amount of GABA [7]. The factors influencing the germination of brown rice include germination temperature, germination time, and the pH of the soaking solution. Yao et al. [8] investigated the effects of different germination conditions on GABA content using Zhongjian 2 as the experimental material. The optimal germination conditions were determined as soaking at 33 °C for 12 h, followed by germination at 35 °C for 26 h, under which conditions the GABA content reached 32.23 mg/100 g. Meanwhile, Zhang et al. [9] optimized the germination process for Jinchuan red brown rice. They discovered that soaking at 25 °C for 2 h, followed by germination at 40 °C for 48 h, resulted in a GABA content of 46.34 mg/100 g.

These findings highlight the potential for enhancing GABA content through optimized germination processes, contributing to the development of functional rice varieties rich in this beneficial amino acid. The GABA content of white glutinous rice, fragrant glutinous rice, and giant embryo glutinous rice was significantly higher than that in regular white rice. Mid- and late-maturing varieties also exhibited markedly higher GABA levels compared to early-maturing ones [10,11]. This variation in GABA content among different germplasms is linked to grain traits and internal anabolism. Giant embryo rice, in particular, has an embryo that is 2–3 times larger than that of regular rice, resulting in significantly higher levels of protein, calcium, phosphorus, and dietary fiber [12]. In the amino acid metabolic pathway, glutamate (Glu) is converted into GABA through the action of glutamate decarboxylase. Similarly, in the polyamine degradation pathway, 4-aminobutyraldehyde is transformed into GABA by amino acid dehydrogenase [13]. Additionally, incorporating GABA enzymatic reaction substrates such as monosodium glutamate and Glu into the soaking solution can effectively enhance GABA enrichment [14]. Current research on the enrichment of GABA content is primarily focused on one or several rice varieties. However, due to significant variations in GABA content among different rice germplasms, these findings are not universally applicable. Additionally, there is a lack of comprehensive reports on the physiological and biochemical factors influencing GABA content. This study addresses these gaps by examining 225 globally sourced micro-core germplasm materials to identify optimal enrichment conditions for various rice germplasms. By analyzing the correlation between GABA content, grain traits, embryo weight ratio, and the amino acids involved in GABA biosynthesis, this study elucidates the key factors contributing to GABA content variability across different germplasms. The findings provide a theoretical foundation for developing GABA enrichment methods tailored to diverse rice germplasms. Furthermore, this research lays the groundwork for the screening of superior GABA-rich rice germplasms.

## 2. Results and Analysis

### 2.1. Effect of Germination Time on GABA Content

**Figure 1:**
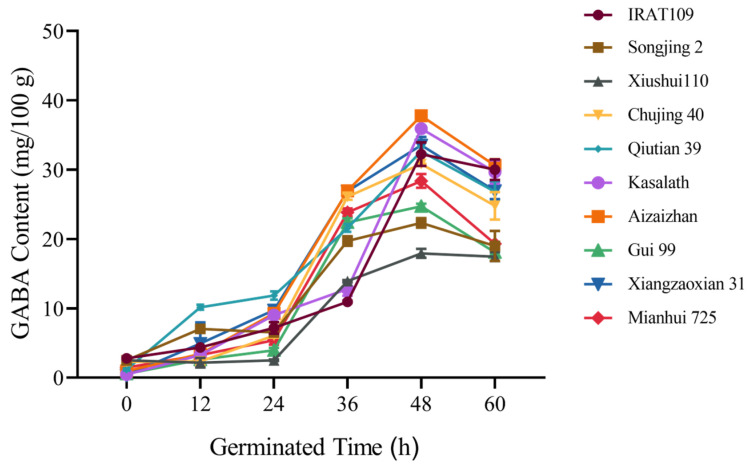
Effect of different germination times on GABA content in ten rice varieties. Data are presented as mean ± standard deviation (*n* = 3).

Five samples each of *indica* rice and *japonica* rice were randomly selected from the 225 rice germplasm resources (Figure 1). The results revealed that the GABA content in all rice varieties increased consistently during the first 48 h of germination, except for Songjing 2 which exhibited a transient decrease at 24 h. However, the GABA content in all varieties began to decrease between 48 and 60 h of germination. Among the ten varieties tested, Aizaizhan had the highest GABA content at 48 h, reaching 37.76 mg/100 g. The results indicated that for the majority of the tested rice germplasms, the optimal germination time to maximize GABA content is 48 h.

### 2.2. Effect of Germination Temperature on GABA Content

**Figure 2:**
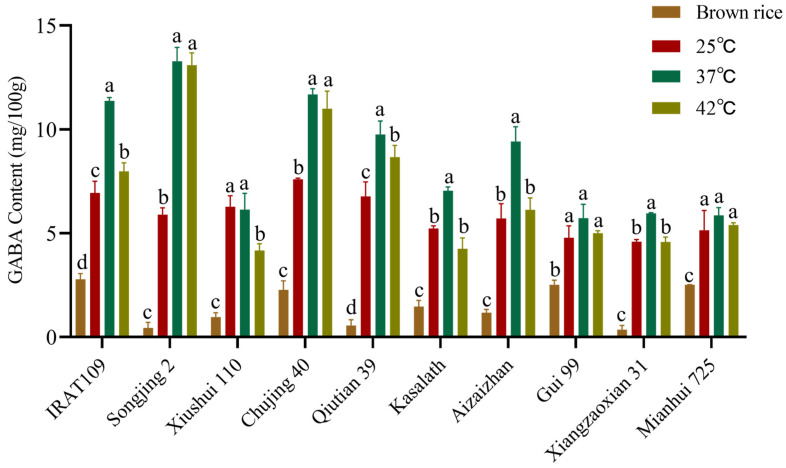
The effect of different germination temperatures on GABA content in ten rice varieties. The statistically significant differences are indicated by a different letter (*p* < 0.05) above each of the different colored columns of each presented histogram. Statistical data were analyzed using one-way ANOVA and Tukey’s post hoc tests. Data are presented as mean ± standard deviation (*n* = 3).

The GABA content of the varieties in Section 2.1 was measured at different temperatures (25 °C, 37 °C, and 42 °C) after 48 h of germination. The results showed a significant increase in GABA content across all samples within the tested temperature range (Figure 2). Among the tested varieties, all except Xiushui 110 reached their peak GABA content at a germination temperature of 37 °C. Among them, the GABA content of Songjing 2 was the highest at 37 °C, reaching 13.28 mg/100 g, which was 30.0 times that of brown rice. When germinated at 37 °C, GABA content increased significantly compared to brown rice (*p* < 0.05): IRAT 109 (4.0-fold), Chujing 40 (5.1-fold), Qiutian 39 (17.5-fold), Kasalath (4.8-fold), Aizaizhan (8.0-fold), Gui 99 (2.2-fold), Xiangzaoxian 31 (16.6-fold) and Mianhui 725 (2.3-fold). Therefore, the optimal germination temperature for different rice varieties was 37 °C.

### 2.3. Effect of pH on GABA Content

**Figure 3:**
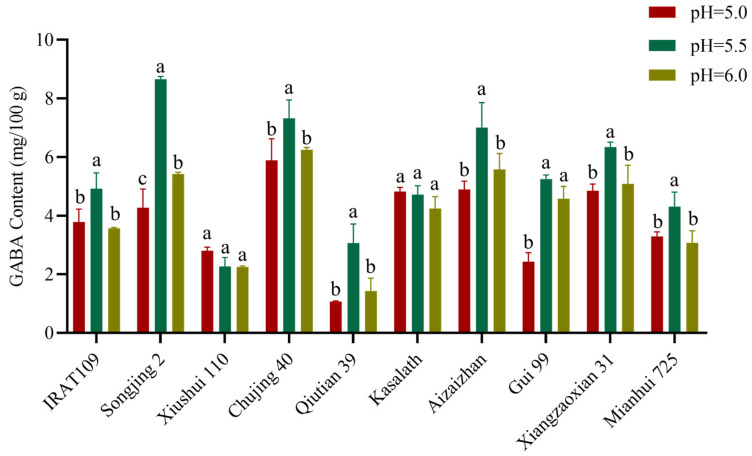
The effect of pH on GABA content in ten rice varieties. The statistically significant differences are indicated by a different letter (*p* < 0.05) above each of the different colored columns of each presented histogram. Statistical data were analyzed using one-way ANOVA and Tukey’s post hoc tests. Data are presented as mean ± standard deviation (*n* = 3).

The GABA content of the varieties in Section 2.1 was measured at different pH levels of the soaking solution after 48 h of germination at 37 °C. The results showed a significant increase in GABA content across all varieties within the tested pH range (Figure 3). Among the tested varieties, Xiushui 110 and Kasalath showed no significant difference in GABA content across the three pH conditions. For Gui 99, no significant difference was observed in GABA content between a pH of 5.5 and 6.0. However, both pH levels resulted in significantly higher GABA content (2.15-fold and 1.88-fold, respectively) compared to a pH of 5.0 (*p* < 0.05). The remaining seven varieties exhibited significantly higher GABA content at a pH of 5.5 compared to other pH conditions. Specifically, compared to a pH of 5.0, the increases in GABA content were significant (*p* < 0.05), with fold changes as follows: IRAT 109 (1.29-fold), Songjing 2 (2.02-fold), Chujing 40 (1.24-fold), Qiutian 39 (2.86-fold), Aizaizhan (1.43-fold), Xiangzaoxian 31 (1.30-fold), and Mianhui 725 (1.30-fold). Similarly, compared to a pH of 6.0, the enhancements remained significant (*p* < 0.05): IRAT 109 (1.37-fold), Songjing 2 (1.59-fold), Chujing 40 (1.17-fold), Qiutian 39 (2.14-fold), Aizaizhan (1.25-fold), Xiangzaoxian 31 (1.24-fold), and Mianhui 725 (1.40-fold). It was demonstrated that the pH level of the soaking solution plays a crucial role in GABA synthesis in rice germplasms. Specifically, a soaking solution with a pH of 5.5 was identified as the optimal condition for enhancing GABA content in various rice varieties. Furthermore, the optimal conditions for GABA enrichment across rice genotypes were germination at 37 °C in a pH of 5.5 solution for 48 h.

### 2.4. Analysis of the Difference of GABA Content Among Different Rice Germplasm Resources

We quantified the GABA content in 225 rice varieties under optimal conditions (germination conditions: temperature, 37 °C; germination time, 48 h; the pH of soaking solution, 5.5). The results showed significant variation in GABA content, ranging from 0.49 mg/100 g to 39.24 mg/100 g, with a coefficient of variation (CV) of 61.31%. This indicated considerable diversity among the experimental varieties. Among the varieties, Jefferson had the highest GABA content at 39.24 mg/100 g, while Peiai 64 had the lowest at 0.49 mg/100 g. The average GABA content across all varieties was 9.11 mg/100 g. Approximately 67% of the rice varieties had GABA content in the range of 5 to 15 mg/100 g, while only 1.3% exhibited GABA levels exceeding 30 mg/100 g. The top 10 rice varieties with the highest GABA content, as measured by this method, were Jefferson, GIZA 178, Suishashani, KON SUITO5, WIR 1021, C101A51 (Pi2), Taitougu, Jamac, Jia 45, and Liantangzao. These included eight *indica* rice varieties and two *japonica* rice varieties. Conversely, the 10 varieties with the lowest GABA content were Sunbonnet, Yugu, Xindao 11, Longjing 21, Gumei 4, Zhongzhe B, Erjiuqing, Zhong R8006, Zhongjian 2, and Peiai 64, comprising eight *indica* rice and two *japonica* rice varieties (Table 1).

### 2.5. Correlation Analysis of GABA Content in Germinated Brown Rice with Brown Rice Morphology and Rice Embryo Traits

**Figure 4:**
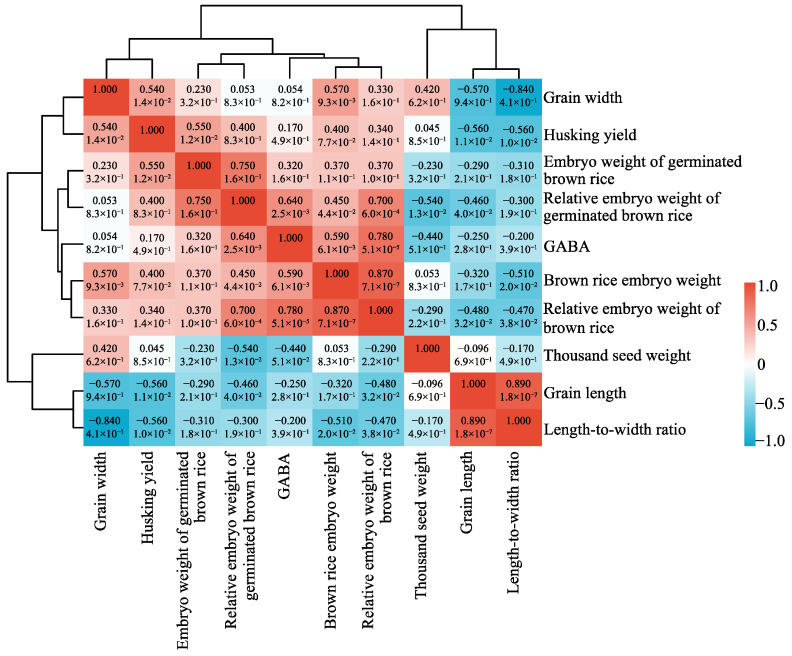
Correlation analysis of GABA content with brown rice morphology. The numbers in the color blocks represent the Pearson correlation coefficients (*r*) and their corresponding *p*-values (*p* < 0.05). Color intensity reflects the strength of the correlation (red: positive; blue: negative) (*n* = 20).

Based on the findings from Section 2.4, which revealed significant variations in the GABA content among different germplasms, we selected the 10 most contrasting accessions from each extreme (high- and low-GABA groups) for the subsequent analysis of the key determinants underlying GABA content variation. We analyzed their correlation with brown rice morphology and embryo traits (Figure 4). The results indicated a strong positive correlation between the GABA content and the embryo weight of brown rice, both the relative and absolute embryo weight (*p* < 0.01). Furthermore, a strong positive correlation was also observed between the GABA content and the relative embryo weight of germinated brown rice (*p* < 0.05). Notably, the correlation between GABA content and the relative embryo weight of brown rice was highest at 0.78, while correlations for the other two traits were recorded at 0.59 and 0.64, respectively. Furthermore, the GABA content demonstrated a weak positive correlation with grain width, husking yield, and germinated brown rice embryo weight. Conversely, it exhibited negative correlations with thousand-seed weight, grain length, and length-to-width ratio. The research results indicated that brown rice varieties with a higher relative embryo weight tend to exhibit increased GABA content.

### 2.6. Relationship Between GABA Content and Amino Acid Content

**Figure 5:**
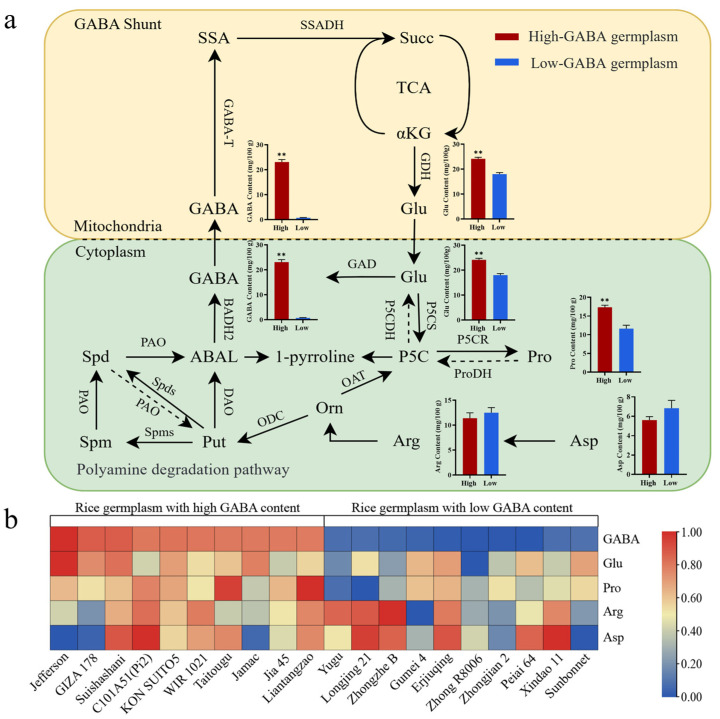
The metabolic pathways of GABA within organisms and the differences in the contents of GABA related amino acids between high-GABA varieties and low-GABA varieties. (**a**) GABA synthesis metabolic pathway; (**b**) differences in GABA and its metabolically related amino acids contents between 10 high-GABA varieties and 10 low-GABA varieties. SSA: succinic acid; SSADH: succinate dehydrogenase; Succ: succinic acid; GABA-T: γ-aminobutyric acid transaminase; αKG: α-ketoglutaric acid; GDH: glutamate dehydrogenase; GAD: glutamate decarboxylase; BADH2: betaine aldehyde dehydrogenase; P5CDH: Δ^1^-pyrroline-5-carboxylate dehydrogenase; P5CS: Δ1-pyrroline-5-carboxylate dehydrogenase; Spd: spermidine; PAO: polyamine oxidase; ABAL: 4-aminobutyraldehyde; P5C: e, Δ1-pyrroline-5-carboxylic acid; ProDH: proline dehydrogenase; P5CR: Δ1-pyrroline-5-carboxylic acid reductase; Spds: spermidine synthase; DAO: diamine oxidase; OAT: ornithine aminotransferase; Spm: spermine; Spms: spermine synthase; Put: putrescine; ODC: ornithine decarboxylase; Orn: ornithine. The heat map was processed by row standardization. Data are presented as mean ± standard deviation (*n* = 3), ** *p* < 0.01 compared with minimum, Student’s *t*-test.

To identify factors underlying GABA variation, we analyzed amino acid contents in the GABA pathway using 20 extreme varieties from Section 2.4 (Figure 5). Compared to low-GABA germplasm materials, which contained an average of 18.60 mg/100 g of Glu and 11.50 mg/100 g of proline (Pro), the high-GABA germplasm materials had significantly higher averages of 24.80 mg/100 g of Glu and 17.30 mg/100 g of Pro. The average Glu and Pro contents in the high-GABA germplasm materials were 1.34 and 1.31 times higher, respectively, than those in the low-GABA germplasm materials (Figure 5b). In the GABA metabolic pathway, Glu serves as the direct precursor for GABA biosynthesis, while Pro is one of the upstream precursors involved in Glu synthesis [15]. Although arginine (Arg) and aspartate (Asp) are also involved in GABA-related metabolic pathways, their influence on GABA content is relatively limited due to the extended synthetic routes involving intermediate compounds such as ornithine and Δ^1^-pyrroline-5-carboxylate (P5C) (Figure 5a). For Arg and Asp, the average contents in high-GABA germplasm materials were 11.10 mg/100 g and 5.90 mg/100 g, respectively, compared to 12.30 mg/100 g and 6.70 mg/100 g in low-GABA germplasm materials (Figure 5b). Data analysis confirmed that the Arg and Asp contents showed no significant intergroup variation. Notably, Glu and Pro levels correlated positively with GABA accumulation. Conversely, Arg and Asp fluctuations demonstrated no meaningful correlation with GABA dynamics.

**Figure 6:**
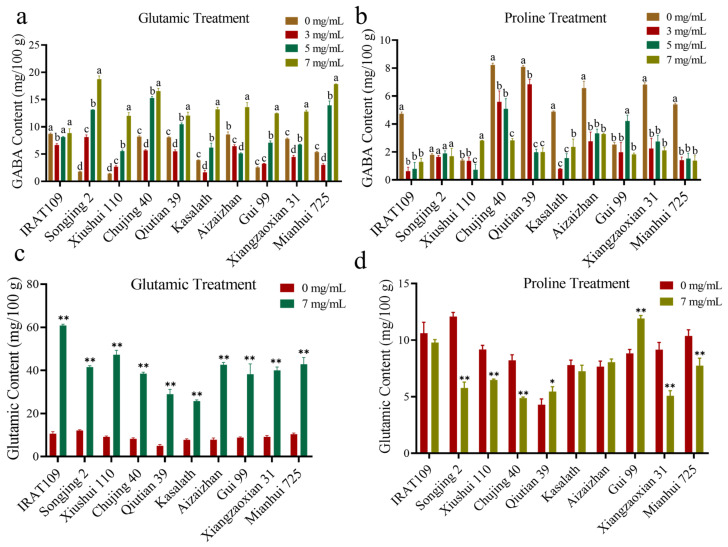
The content of GABA and Glu in 10 rice varieties under different concentrations of Glu and Pro. Variation in GABA content among 10 rice varieties under different concentrations of Glu (**a**) and Pro (**b**). Statistical data were analyzed using one-way ANOVA and Tukey’s post hoc tests. Data are presented as mean ± standard deviation (*n* = 3). The statistically significant differences were indicated by a different letter (*p* < 0.05) above each of the different colored columns of each presented histogram. Variation in Glu content (**c**) and Pro content (**d**) among 10 rice varieties under exogenous Glu concentrations of 0 mg/mL and 7 mg/mL. Data are presented as mean ± standard deviation (*n* = 3), * *p* < 0.05, ** *p* < 0.01 compared with the control (0 mg/mL), Student’s *t*-test.

Different concentrations of Glu and Pro were applied to the 10 rice varieties detailed in Section 2.4, to investigate the effects of these amino acids on the GABA content of rice with different genotypes (Figure 6). The results showed that under single Glu application, the GABA content of germinated brown rice generally increased with higher Glu concentrations, except for Aizaizhan. For most materials, GABA content decreased at a Glu concentration of 3 mg/mL but then increased as the concentration rose further. Notably, Songjing 2 achieved its highest GABA content of 18.70 mg/100 g at a Glu concentration of 7 mg/mL, which was 10.4 times higher than that of the untreated sample (Figure 6a). In contrast, when Pro was applied alone, all materials exhibited either a decreasing trend in GABA content or no significant difference as the concentration increased (Figure 6b). Subsequently, further measurements were taken of the Glu content in germinated brown rice under conditions where both the Glu and Pro concentrations were set at 7 mg/mL. The results showed that under Glu treatment, the internal Glu content in germinated brown rice significantly increased (*p* < 0.01). Specifically, IRAT 109 exhibited the highest Glu content at 60.80 mg/100 g, which was 5.9 times higher than that of untreated germinated brown rice (Figure 6c). Under Pro treatment, the Glu content in most tested varieties significantly decreased compared to the control. Notably, Songjing 2, Xiushui 110, Chujing 40, Xiangzaoxian 31, and Mianhui 725 showed significant reductions in Glu content (Figure 6d). The results indicated that the internal Glu content of germinated brown rice decreased after external application of Pro, thereby directly affecting GABA synthesis. In summary, during germination, applying Glu significantly increased the internal Glu content in germinated brown rice, enhancing GABA synthesis. Conversely, applying Pro inhibited internal Glu synthesis, thereby reducing GABA content.

## 3. Discussion

Enhancing GABA content in diverse rice cultivars represents a promising strategy to augment their nutritional value, thereby promoting consumer health. GABA synthesis predominantly occurs within the embryonic tissue of the grain [16]. The accumulation rate and quantity of GABA are significantly influenced by germination temperature, duration, and the pH level of the soaking solution. Optimal germination conditions are required for enhancing GABA synthesis in rice [17]. This study employed an indoor physicochemical treatment method to germinate brown rice embryos, aiming to bridge the technological gap in GABA enrichment in rice. Our investigation revealed that the optimal germination conditions encompassed a germination time of 48 h, a temperature of 37 °C, and a soaking solution pH of 5.5. Under these conditions, the GABA content across 225 rice germplasms was assessed (Figure 1, Figure 2 and Figure 3). Notably, the Jefferson cultivar exhibited the highest GABA concentration, which was 11.2-fold higher than that of untreated brown rice. This finding underscores the potential of these conditions to substantially elevate GABA levels in rice. Subsequent analyses demonstrated significant variability in GABA content among different rice cultivars (Table 1). As is well known, Glu, the direct substrate for GABA synthesis, is the most important and fundamental raw material in the process. The conversion of Glu to GABA requires the catalytic action of glutamate decarboxylase (GAD), a process that depends on vitamin B6 (pyridoxal phosphate, PLP) as a cofactor to ensure the effective functioning of GADs [18]. In this study, we investigated the correlation between GABA content and grain traits, as well as the amino content within the synthetic metabolic pathway. Our findings reveal that rice varieties with higher relative embryo weight and elevated levels of Glu and Pro exhibit significantly increased GABA content. Increased levels of Glu and Pro indicate a more sufficient supply of precursors for GABA synthesis. Moreover, during the germination process, the activities of GABA-related enzymes, such as GAD, proline dehydrogenase (ProDH), and Δ^1^-pyrroline-5-carboxylate dehydrogenase (P5CDH), are significantly enhanced, thereby promoting the accumulation of GABA. The results of this study further confirmed that the exogenous application of Glu markedly elevated GABA accumulation. These results underscore the critical role of Glu and Pro in GABA biosynthesis and highlight the potential for enhancing GABA content through physiological and biochemical manipulation. The observed relationship between embryo weight and GABA concentration suggests that selection for larger embryos may offer a viable strategy for improving the nutritional profile of rice grains (Figure 4). Zhang et al. [19] germinated Early 944 at 30% moisture and 30 °C for 48 h, achieving a GABA content of 39.21 mg/100 g, 1.5 times that of brown rice. Dongnong 429 treated with 50 mg/mL cellulase for 90 min followed by germination at 30 °C for 32 h resulted in 32 mg/100 g GABA, 7.5 times higher than brown rice [20]. While previous studies have also used germination to enhance GABA, their scope has been limited to single varieties under specific conditions. In contrast, our study analyzed 10 diverse rice accessions from a global microcore collection of 225 germplasms, providing broader representativeness and mechanistic insights into GABA variation. Based on these findings, it was determined that embryo weight and internal Glu levels were important factors influencing GABA content in brown rice. These findings provide a basis for enhancing the nutritional quality and functional attributes of rice.

The GABA metabolic pathway involves amino acids such as Glu, Pro, Arg, and Asp, influencing GABA synthesis directly or indirectly. Post germination, brown rice exhibits a sharp increase in Glu and Asp levels, boosting total free amino-acid content. Varietal differences are notable [21,22]. This study analyzed 20 rice germplasm samples revealing positive correlations among GABA, Glu, and Pro in germinated brown rice (Figure 5). This suggests potential intercorrelations among these amino acids. Conversely, Arg and Asp metabolism yields putrescine (Put) and 4-aminobutyraldehyde (ABAL), possibly causing insignificant correlations with GABA levels. Exogenous Glu application markedly elevates GABA content in germinated brown rice by converting to GABA via GAD activity (Figure 6a). Changes in Glu levels directly impact the GABA concentration in seeds. Previous studies have shown that the application of sodium glutamate and controlled humidity can increase GABA levels in germinated brown rice [23,24]. This effect was likely due to the influence of Glu on the synthesis of GABA. Pro is oxidized by ProDH in mitochondria to produce P5C, which is subsequently converted into Glu by P5CDH [15]. ProDH is a key enzyme responsible for the degradation of Pro in higher plants. However, the exogenous application of Pro significantly reduced the GABA content in germinated brown rice (Figure 6b), which contrasts with the results observed in the earlier experiment (Figure 5). The observed difference may result from the exogenous application of Pro, which can modulate the expression of key genes such as *P5CS* and *ProDH* and induce ROS (reactive oxygen species) imbalance [25]. This imbalance may deplete Glu in grains, thereby reducing GABA content (Figure 6d). However, the precise mechanisms underlying this phenomenon require further investigation. In contrast, under normal conditions in the earlier experiment, sufficient endogenous Pro functions as a feedback regulator, inhibiting the expression of *P5CS* while inducing the expression of *ProDH* [26]. This regulation led to the accumulation of Glu, which in turn increased the GABA content.

This study utilized indoor physicochemical treatment methods to determine the optimal enrichment conditions, thereby increasing the GABA content in various rice germplasms. Nonetheless, the elevation of GABA content is contingent upon the foundational germplasm, with the pivotal factor being the advancement in GABA-specific germplasm. By understanding the potential factors that influence GABA accumulation, we can develop effective strategies to enhance GABA content in rice. This will benefit both agricultural productivity and human health. Japanese scholars Satoh and Omura [27] were the first to successfully obtain a large-embryo mutant of ‘Kinmaze’ rice using mutagenesis techniques. Building on this, Maeda et al. [28] used ‘Kinmaze’ as a parent line, and developed a new large-embryo rice variety named ‘Haiminori’ through hybridization and systematic selection in 2001. ‘Haiminori’ exhibits significantly higher GABA content compared to other rice varieties. Since then, breeding efforts have gradually yielded successful outcomes, focusing on large-embryo rice. Khwancha et al. [29] discovered that there is also genetic diversity in the GABA content of germinated brown rice from different Thai germplasm. The GABA content in germinated brown rice “CN1” was the highest (401.70 μg/g), significantly higher than that of other varieties (71.10–156.20 μg/g). The accumulation of GABA in rice is influenced not only by genetic regulatory factors but also by external environmental factors. In other species, the GABA content in edible mushroom fruiting bodies is influenced by the nitrogen content of the culture medium, the type of nitrogen source, and the cultivation time [30]. During the growth of rice, environmental factors such as temperature, humidity, and soil conditions may also affect the biosynthesis of GABA. Integrating molecular breeding with advanced modern cultivation techniques is expected to significantly increase the GABA content.

Future advances may enable the isolation and cloning of key GABA synthesis genes through screening and precise identification of GABA-rich germplasm. The GABA biosynthesis pathway is controlled by multiple genes. To date, approximately 20 genes involved in the GABA metabolic pathway have been identified, including the key GABA synthesis genes (*GADs*) and the catabolism gene *BADH2* [31]. Genes involved in GABA synthesis and metabolism can be precisely targeted and edited using CRISPR-Cas9 technology, thereby enhancing rice quality and GABA content. Targeted genetic improvements can lead to the development of new functional rice varieties, enhancing their nutritional profiles. By developing rice with elevated GABA levels, we can meet diverse nutritional needs, thereby increasing the value of rice as a staple food. This strategy promises significant contributions to public health by improving dietary quality. Such innovations may revolutionize rice cultivation, offering tailored solutions to address specific nutritional deficiencies. Consequently, these efforts have the potential to elevate overall population health, making rice a more potent vehicle for delivering essential nutrients. Thus, advancing genetic research in this domain remains crucial for achieving sustainable and nutritious food systems.

## 4. Materials and Methods

### 4.1. Plant Materials and Growth Conditions

A total of 89 *japonica* and 136 *indica* rice accessions were selected and planted at the Fuyang base of the China Rice Research Institute in Hangzhou (30°04′ N, 119°55′ E), Zhejiang Province, China in 2022, under natural conditions. Each variety was grown in four rows of six plants each, maintaining a uniform spacing of 20 × 20 cm, with individual transplantation. All materials received standard agronomic management practices throughout the growing period, including disease and pest controls and moderate fertility management.

### 4.2. Germination Conditions and GABA Quantification

Five representative varieties of *indica* rice (Kasalath, Aizaizhan, Gui 99, Xiangzaoxian 31, Mianhui 725) and *japonica* rice (IRAT 109, Songjing 2, Xiushui 110, Chujing 40, Qiutian 39) were randomly selected. Intact brown rice grains were surface-sterilized with 1% (*v*/*v*) sodium hypochlorite (NaClO, Xilong Scientific, Shantou, China) for 15 min, followed by triple rinsing with deionized water. The disinfected brown rice was then soaked in a phosphate buffer solution (Labgic Technology, Beijing, China) with an ionic concentration of 0.1 mol/L (pH 5.0, 5.5, or 6.0) in a constant-temperature water bath (Junsi Brand, Jiaxing, China) for 24 h. After soaking, the seeds were thoroughly washed and placed on filter paper-lined petri dishes. Incubation was carried out in a constant-temperature drying oven (Jingxin Brand, Shanghai, China) at varying temperatures (25 °C, 37 °C, and 42 °C) and for different durations (12, 24, 36, 48, or 60 h). During this period, moisture was maintained by spraying a fixed amount of deionized water every 6 h, and the brown rice was periodically turned to ensure uniform humidity. Upon completion of the germination process, the germinated seeds were dried and prepared for GABA content measurement.

GABA extraction was performed by mixing rice flour (0.08 g) with distilled water at a solid-to-liquid ratio of 1:20 (g/mL), based on the method optimized by Fan et al. [32]. The mixture was placed in a 60 °C constant-temperature water bath with shaking every 15 min for 2 h. After incubation, the mixture was centrifuged at 11,000 rpm for 10 min. The supernatant (1 mL) was transferred to a centrifuge tube. The color reaction was performed according to the method described by Yu et al. [33], and absorbance was measured at 630 nm using a microplate reader (TECAN Infinite 200 PRO, Männedorf, Switzerland).

### 4.3. Determination of Grain Traits and Relative Embryo Weight

Based on the optimal enrichment conditions established in Section 4.3, we germinated 225 rice materials. From these, we selected the 10 varieties with the highest and lowest GABA content for determining grain traits, husking yield, thousand-grain weight, and relative embryo weight (Table A1).

Determination of rice grain traits, husking yield, and thousand-seed weight: The grain length and width of three groups of 100 seeds were measured using a vernier caliper (Guanglu Brand, Guangxi, China), and the average values were used to calculate the length-to-width ratio. For each variety, three samples of 20 g of seeds were prepared. Seeds were dehulled using a B-45 huller (Liangan Brand, Shandong, China) to obtain brown rice, which was then weighed. The husking yield was calculated according to the method by Yuan [34]. The thousand-grain weight was determined using SC-G seed analyzer (Wanshen Brand, Hangzhou, China).

HY% = BR/PR·100 (HY: husking yield; BR: brown rice weight, g; PR: paddy rice weight, g)

LWR = GL/GW (LWR: length-to-width ratio; GL: grain length, mm; GW: grain width, mm)

Determination of embryo weight of brown rice: A total of 3 groups of 100 plump brown rice seeds were weighed, and the weight was recorded. After removing the embryo from the brown rice, the seeds were weighed again to calculate the embryo weight and relative embryo weight.

EBR = BR − DBR (EBR: brown rice embryo weight, g; DBR: de-embryo weight of brown rice, g)

REBR = EBR/BR (REBR: relative embryo weight of brown rice)

Determination of the embryo weight of germinated brown rice: Similarly, three groups of 100 plump brown rice seeds were selected for germination treatment. After germinating for 6 h, the seeds were washed, dried, and weighed. The embryo was then removed from the germinated brown rice and weighed again to calculate the embryo weight and relative embryo weight.

EGR = GR − DGR (EGR: embryo weight of germinated brown rice, g; GR: germinated brown rice weight, g; DGR: de-embryo weight of germinated brown rice, g)

REGR = EGR/GR (REGR: relative embryo weight of germinated brown rice)

### 4.4. Determination of the Content of Amino Acids

#### 4.4.1. Determination of Amino Acids in Rice

Under the optimal enrichment conditions established in Section 4.3, the contents of Glu, Pro, Arg, and Asp were determined in 20 selected materials using an amino acid content determination kit (Grace Biotechnology, Suzhou, China).

#### 4.4.2. External Application of Different Concentrations of Amino Acids

The germination procedure followed Section 4.2. Seeds were soaked in Glu or Pro solutions at three concentrations (3, 5, and 7 mg/mL). During germination, the corresponding amino acid solution was sprayed every 6 h to maintain concentration and measured the GABA content after completion.

### 4.5. Statistical Analysis

Statistical analyses and data visualization were performed using R program (version 4.1.2) and Microsoft Excel. Data are presented as mean ± SD based on a minimum of three biological replicates. Significance was assessed using Student’s *t*-test or one-way ANOVA with Tukey’s post hoc test. Correlation heatmaps were generated with TBtools (version 2.142).

## 5. Conclusions

Based on the aforementioned findings, the optimal conditions for GABA enrichment were determined, which systematically elucidated the factors affecting the GABA content variation in rice germplasms from both intrinsic and extrinsic perspectives. Maximizing GABA content in germinated brown rice required soaking at a pH of 5.5 in 0.1 mol/L phosphate buffer, followed by germination at 37 °C for 48 h. The key physiological and biochemical factors identified were embryo size in brown rice, alongside Glu and Pro concentrations in germinated brown rice. Germplasms with larger embryos and higher Glu and Pro levels exhibited greater GABA accumulation. This study provides insights for developing GABA-enriched rice products and offers valuable resources for breeding rice varieties with enhanced GABA content.

## Figures and Tables

**Table 1:** The 20 rice varieties with the highest and lowest GABA content.

Variety Name	Type	Content (mg/100 g)	Variety Name	Type	Content (mg/100 g)
Jefferson	*Indica*	39.24 ± 3.87	Sunbonnet	*Indica*	1.00 ± 0.31
GIZA 178	*Indica*	25.82 ± 1.48	Yugu	*Indica*	0.95 ± 0.12
Suishashani	*Japonica*	25.76 ± 4.89	Xindao 11	*Japonica*	0.95 ± 0.34
KON SUITO5	*Indica*	20.90 ± 2.57	Longjing 21	*Japonica*	0.93 ± 0.21
WIR 1021	*Indica*	20.50 ± 0.91	Zhongzhe B	*Indica*	0.83 ± 0.26
C101A51 (Pi2)	*Indica*	20.10 ± 0.93	Gumei 4	*Indica*	0.73 ± 0.09
Taitougu	*Indica*	19.90 ± 1.48	Erjiuqing	*Indica*	0.59 ± 0.04
Jamac	*Indica*	19.90 ± 2.61	Zhong R8006	*Indica*	0.52 ± 0.11
Jia 45	*Japonica*	19.70 ± 4.15	Zhongjian 2	*Indica*	0.52 ± 0.13
Liantangzao	*Indica*	19.40 ± 2.77	Peiai 64	*Indica*	0.49 ± 0.04

Data are presented as mean ± standard deviation (*n* = 3).

## Data Availability

Data are contained within the article.

## Appendix A

**Table A1:** Grain and embryo characteristics of 20 rice varieties.

Variety Name	Grain Length	Grain Width	Length-to-Width Ratio	GABA	Thousand-Seed Weight	Brown Rice Embryo Weight	Relative Embryo Weight of Brown Rice	Embryo Weight of Germinated Brown Rice	Relative Embryo Weight of Germinated Brown Rice	Husking Yield
Jefferson	7.377 ± 0.210	2.245 ± 0.081	3.288 ± 0.091	39.240	21.159 ± 2.311	0.019 ± 0.002	0.032 ± 0.002	0.074 ± 0.003	0.094 ± 0.004	32.105 ± 3.210
GIZA178	5.131 ± 0.150	2.319 ± 0.081	2.215 ± 0.113	25.820	17.551 ± 0.522	0.016 ± 0.001	0.033 ± 0.002	0.096 ± 0.002	0.147 ± 0.002	56.992 ± 4.111
Suishashani	5.918 ± 0.270	2.144 ± 0.084	2.762 ± 0.127	25.760	19.410 ± 1.702	0.022 ± 0.002	0.043 ± 0.003	0.073 ± 0.003	0.115 ± 0.005	32.59 ± 2.796
C101A51(Pi2)	5.266 ± 0.230	2.496 ± 0.103	2.112 ± 0.094	20.110	19.700 ± 0.433	0.018 ± 0.001	0.032 ± 0.001	0.076 ± 0.002	0.103 ± 0.002	59.638 ± 4.762
KON SUIT05	6.163 ± 0.160	2.686 ± 0.083	2.296 ± 0.080	20.900	21.412 ± 1.171	0.025 ± 0.001	0.038 ± 0.002	0.090 ± 0.002	0.115 ± 0.003	66.631 ± 4.732
WIR1021	6.297 ± 0.220	2.378 ± 0.074	2.650 ± 0.101	20.500	19.310 ± 1.420	0.026 ± 0.001	0.043 ± 0.001	0.133 ± 0.007	0.161 ± 0.009	49.359 ± 3.72
Taitougu	6.250 ± 0.270	1.961 ± 0.066	3.189 ± 0.156	19.960	16.750 ± 0.710	0.016 ± 0.001	0.030 ± 0.003	0.093 ± 0.009	0.143 ± 0.015	50.905 ± 4.350
Jamac	7.367 ± 0.200	2.677 ± 0.063	2.754 ± 0.091	19.900	21.487 ± 1.582	0.024 ± 0.002	0.032 ± 0.003	0.092 ± 0.004	0.099 ± 0.003	58.023 ± 3.240
Jia 45	5.174 ± 0.100	2.837 ± 0.091	1.825 ± 0.067	19.740	22.593 ± 1.01	0.024 ± 0.002	0.035 ± 0.003	0.095 ± 0.002	0.106 ± 0.002	70.239 ± 4.604
Liantangzao	5.669 ± 0.220	2.659 ± 0.091	2.136 ± 0.130	19.450	21.044 ± 1.555	0.020 ± 0.001	0.033 ± 0.002	0.109 ± 0.008	0.136 ± 0.011	49.713 ± 4.823
Yugu	8.305 ± 0.260	2.008 ± 0.058	4.140 ± 0.175	0.950	21.809 ± 1.186	0.009 ± 0.001	0.012 ± 0.001	0.069 ± 0.009	0.076 ± 0.004	5.448 ± 0.980
Longjing21	5.531 ± 0.220	2.753 ± 0.103	2.012 ± 0.102	0.930	25.310 ± 3.390	0.019 ± 0.002	0.028 ± 0.003	0.068 ± 0.003	0.079 ± 0.005	53.810 ± 3.651
Zhongzhe B	6.945 ± 0.230	2.284 ± 0.094	3.047 ± 0.180	0.830	23.287 ± 1.511	0.019 ± 0.001	0.026 ± 0.001	0.096 ± 0.002	0.101 ± 0.002	59.853 ± 3.990
Gumei4	5.914 ± 0.160	2.809 ± 0.102	2.108 ± 0.092	0.730	21.443 ± 2.721	0.017 ± 0.001	0.024 ± 0.001	0.075 ± 0.001	0.077 ± 0.001	40.989 ± 4.780
Erjiuqing	5.492 ± 0.24	2.570 ± 0.073	2.138 ± 0.102	0.590	22.813 ± 0.511	0.015 ± 0.001	0.024 ± 0.002	0.090 ± 0.004	0.111 ± 0.003	53.857 ± 2.691
ZhongR8006	7.500 ± 0.20	2.063 ± 0.042	3.636 ± 0.114	0.520	22.846 ± 1.271	0.009 ± 0.001	0.014 ± 0.003	0.077 ± 0.001	0.091 ± 0.001	34.511 ± 3.282
Zhongjian2	9.607 ± 0.27	2.268 ± 0.082	4.277 ± 0.181	0.520	21.227 ± 0.45	0.011 ± 0.001	0.016 ± 0.001	0.057 ± 0.003	0.066 ± 0.004	12.881 ± 1.345
Peiai64	6.569 ± 0.22	1.991 ± 0.068	3.302 ± 0.121	0.490	18.465 ± 1.37	0.014 ± 0.002	0.026 ± 0.003	0.060 ± 0.001	0.088 ± 0.002	43.909 ± 4.791
Xindao11	5.620 ± 0.18	2.508 ± 0.101	2.243 ± 0.081	0.950	20.196 ± 0.946	0.017 ± 0.002	0.026 ± 0.002	0.094 ± 0.009	0.102 ± 0.022	74.154 ± 4.346
Sunbonnet	7.267 ± 0.31	2.251 ± 0.121	3.238 ± 0.231	1.000	18.319 ± 1.324	0.012 ± 0.001	0.017 ± 0.002	0.101 ± 0.005	0.113 ± 0.006	57.877 ± 3.482

Data are presented as mean ± standard deviation (*n* ≥ 3).

## References

[1] Ngo D.H., Vo T.S. (2019). An updated review on pharmaceutical properties of Gamma-Aminobutyric Acid. Molecules.

[2] Kim S., Jo K., Hong K.B., Han S.H., Suh H. (2019). GABA and l-theanine mixture decreases sleep latency and improves NREM sleep. Pharm. Biol..

[3] Wu F., Yang N., Touré A., Jin Z., Xu X. (2013). Germinated brown rice and its role in human health. Crit. Rev. Food Sci. Nutr..

[4] Tufail T., Ain H.B.U., Virk M.S., Ashraf J., Ahmed Z., Khalil A.A., Rasheed A., Xu B. (2025). GABA (γ-aminobutyric acid) enrichment and detection methods in cereals: Unlocking sustainable health benefits. Food Chem..

[5] Chinma C.E., Adedeji O.E., Jolayemi O.S., Ezeocha V.C., Ilowefah M.A., Rosell C.M., Adebo J.A., Wilkin J.D., Adebo O.A. (2024). Impact of germination on the techno-functional properties, nutritional composition, and health-promoting compounds of brown rice and its products: A review. J. Food Sci..

[6] Hou D., Tang J., Feng Q., Niu Z., Shen Q., Wang L., Zhou S. (2024). Gamma-aminobutyric acid (GABA): A comprehensive review of dietary sources, enrichment technologies, processing effects, health benefits, and its applications. Crit. Rev. Food Sci. Nutr..

[7] Saikusa T., Okada T., Murai H., Ohmori M., Mori Y., Horino T., Itou M., Onoda A. (2001). The effect of defatting with organic solvent on accumulation of γ-aminobutyric acid (GABA) in the rice germ. J. Jpn. Soc. Food Sci..

[8] Yao S., Zheng L., Zhao S., Xiong S. (2006). Effect of germination conditions on γ-aminobutyric acid content in germinated brown rice. Trans. Chin. Soc. Agric. Eng..

[9] Zhang Y., Zhu Y., Chen J., Xia C., Deng J., Li Y., Li J., Li H., Liu P. (2017). Preparation process of rich GABA germinated red brown rice in Jinchuan. Food Nutr. China.

[10] Bai H., Ma X., Cao G., Liu X., Han L. (2017). Differences in the contents of nutrients and functional components in different types of special rice germplasm. J. Plant Genet. Resour..

[11] Wang Y., Wang Q., Wang H., Sun L., Zhang Y., Tian L., Yang S., Li P. (2016). Screening of γ-aminobutyric acid-rich rice germplasm and its correlation with grain traits. J. Plant Genet. Resour..

[12] Zhang L., Hu P., Tang S., Zhao H., Wu D. (2005). Comparative studies on major nutritional components of rice with a giant embryo and a normal embryo. J. Food Biochem..

[13] Fait A., Fromm H., Walter D., Galili G., Fernie A.R. (2007). Highway or byway: The metabolic role of the GABA shunt in plants. Trends Plant Sci..

[14] Thitinunsomboon S., Keeratipibul S., Boonsiriwit A. (2013). Enhancing gamma-aminobutyric acid content in germinated brown rice by repeated treatment of soaking and incubation. Food Sci. Technol. Int..

[15] Podlešáková K., Ugena L., Spíchal L., Doležal K., Nuria D.D. (2018). Phytohormones and polyamines regulate plant stress responses by altering GABA pathway. New Biotechnol..

[16] Yang R., Geng C., Gu Z. (2016). Activation and tempering on γ-aminobutyric acid accumulation and distribution in brown rice. J. Food Process. Preserv..

[17] Cataldo P.G., Villegas J.M., Giori D.S.G., Saavedra L., Hebert E.M. (2020). Enhancement of γ-aminobutyric acid (GABA) production by *Lactobacillus brevis* CRL 2013 based on carbohydrate fermentation. Int. J. Food Microbiol..

[18] Stover P.J., Field M.S. (2015). Vitamin B-6. Adv. Nutr..

[19] Zhang N., Liu S., Wang L., Pan Q. (2020). Effects of germination and aeration treatment following segmented moisture conditioning on the γ-aminobutyric acid accumulation in germinated brown rice. Int. J. Agric. Biol. Eng..

[20] Zhang Q., Liu N., Wang S., Liu Y., Lan H. (2019). Effects of cyclic cellulase conditioning and germination treatment on the γ-aminobutyric acid content and the cooking and taste qualities of germinated brown rice. Food Chem..

[21] Ohtsubo K., Suzuki K., Yasui Y., Kasumi T. (2005). Bio-functional components in the processed pre-germinated brown rice by a twin-screw extruder. J. Food Compos. Anal..

[22] Chen J., Zhong G., Wang Y., Liu G., Chen W. The variation characteristics of free amino acid content during the germination of different varieties of brown rice. Proceedings of the Sixth Member Conference and Academic Seminar of Guangdong Food Society.

[23] Wang L., Ding G., Li L. (2010). Research progress of proline metabolism. J. Nat. Sci. Harbin Norm. Univ..

[24] Wang Y., Han Y., Gu Z., Li B. (2019). Effects of exogenous monosodium glutamate and ascorbic acid on GABA accumulation in germinated brown rice. Food Chem..

[25] Bauduin S., Latini M., Belleggia I., Migliore M., Biancucci M., Mattioli R., Francioso A., Mosca L., Funck D., Trovato M. (2022). Interplay between proline metabolism and ROS in the fine tuning of root-meristem size in *Arabidopsis*. Plants.

[26] Hayashi F., Ichino T., Osanai M., Wada K. (2000). Oscillation and regulation of proline content by P5CS and ProDH gene expressions in the light/dark cycles in *Arabidopsis thaliana* L.. Plant Cell Physiol..

[27] Satoh H., Omura T. (1981). New endosperm mutations induced by chemical mutagens in rice (*Oryza sativa* L.). Breed. Sci..

[28] Maeda H., Nemoto H., Iida S., Ishii T., Nakagawa N., Hoshino T., Sakai M., Okamoto M., Shinoda H., Yoshida T. (2001). A new rice variety with giant embryos, “Haiminori”. Breed. Sci..

[29] Khwanchai P., Chinprahast N., Pichyangkura R., Chaiwanichsiri S. (2014). Gamma-aminobutyric acid and Glu acid contents, and the GAD activity in germinated brown rice (*Oryza sativa* L.): Effect of rice cultivars. Food Sci. Biotechnol..

[30] Wen Q., Zhao H., Shao Y., Hu Y., Qi Y., Wang F., Shen J. (2023). Determination of γ-aminobutyric acid content and influencing factors of production of major edible mushroom fruiting bodies in China. Mycosystema.

[31] Ma J., Du H., Liu H., Sun Z., Ma W., Ma R., Tian L., Ma T., Li P. (2022). Research progress on γ-aminobutyric acid in rice grains. Hybrid Rice.

[32] Fang Q., Lan Q., Wan L., Cheng J. (2023). System optimization for rapid determination of γ-aminobutyric acid content in brown rice based on Berthelot colorimetric method. Food Nutr. China.

[33] Yu Y., Li M., Li C., Niu M., Dong H., Zhao S., Jia C., Xu Y. (2023). Accelerated accumulation of γ-aminobutyric acid and modifications on its metabolic pathways in black rice grains by germination under cold stress. Foods.

[34] Yuan J. (2022). Study on roughness test of rice yield. J. Chin. Inst. Food Sci. Technol..

